# The Theatre and the Cinema: Some Hygienic Defects, Their Causes and Prevention

**Published:** 1917-09-15

**Authors:** F. Fowell

**Affiliations:** Secretary. Cinematograph Trade Council.


### THE EDITOR'S LETTER-BOX.

### The Theatre and the Cinema: Some Hygienic Defects, their Causes and Prevention.

*To the Editor of* The Hospital.

Sir,—The writer of the article on "The Essential Defects of the Theatre and Cinema" is not quite fair to the latter industry, at any rate, for most of the recent theatres have elaborate heating and ventilating plants installed on the most up-to-date lines. The air is washed, filtered, and warmed or cooled, and changed throughout the hall several times an hour. In one chain of theatres that I know of, the entire contents of the halls are renewed once in every four minutes, and the whole plant of a very costly type was installed under the best expert advice procurable.

But, as you yourself admit, the question of ventilation by no means solves the problem of hygiene in theatres. Apart altogether from the fact that an astonishing number of patrons protest at the very idea of air in motion and assert that they will contract catarrh and colds and rheumatism if they are compelled to "sit in a draught," the fact remains that there are other conditions which simply cannot be nullified without the active co-operation of individual members of the audience. Unfortunately most audiences are not sufficiently alive to the essential condition of clean living to appreciate these points. The person who spits on the carpet is vastly more common—even in better-class seats—than might be imagined, and this is not a matter which a vacuum cleaner can remedy for several hours. The habit of coughing, with or without the shelter of a hand, may not seem very prevalent in a cinema; but it must be remembered that those who cough are, in most cases, the very people whose sputum, projected violently in this way, is most dangerous to those around. As you are aware, experiments have shown that a cough, even without a draught, has been known to project particles of matter over three yards, and effective ventilation may increase this area rather than restrict it. Nor is the matter one which can be dealt with by disinfectants and sterilisation. It would not be impossible to devise a theatre which could be sterilised and disinfected adequately each day, but such a theatre would frankly not be a comfortable one, and these arrangements would not increase the theatre's popularity with most people.

All the difficulties of sheer proximity are questions which the management can hardly deal with from a hygienic point of view. Their theatre may be immaculate when it opens at one o'clock, but by half-past it may, in spite of all his precautions, have been seriously infected. Until individual members of the community have learned to keep their own persons scientifically clean, it is difficult to see how some of the more serious evils of congregation are to be avoided. The problem is not one of the cinemas only; it applies to every place where people congregate, and, in point of fact, probably applies less to the cinemas than to other places by reason of the care taken in ventilation. Some of our churches, especially when open for whole-day services, provide appalling atmospheric conditions, and an analysis of the atmosphere would produce startling results.

If I may venture slightly off my original lines, I would suggest as a layman that some of the worst difficulties of public contagion, especially those relating to nasal and throat infection, are to be found in our larger popular eating-houses. It has been my experience that forks are rarely adequately cleaned, and I do not think they are ever sterilised. Spoons, by reason of their shape, are usually better, but are often in a far from satisfactory condition on busy days. But my chief complaint is in connection with cracked crockery. A chipped cup may pass to and from the lips of fifty or sixty people in the course of a day, and microscopic and bacterial investigation of these dirty brown chip-marks provides a most unpleasant surpise. The process of washing these things in tepid water is probably agreeably stimulating to the degenerate organisms which lurk in them, but does not otherwise disturb them. If one of your investigators will purchase three or four cracked or chipped cups at the nearest restaurant and subject them to a scientific interrogation he will have to thank me for a new thrill.

F. Fowell, Secretary.

Cinematograph Trade Council.

August 21.

[We are, of course, glad to have Mr. Fowell's assurance that the Cinematograph Trade Council is attempting to improve the hygienic conditions of the picture theatres. But we cannot allow that our contributor's article does any injustice to these establishments. Whether we have missed the links of the "chain" of which Mr. Fowell speaks, we cannot say, but not a few experiences have satisfied us that our contributor has dealt moderately with the facts as they actually exist even in some new and fashionable establishments. The continuous performance, involving as it does a constant population in these theatres, is doubtless a special difficulty from the point of view of ventilation and of hygiene. We recognise this fully, and indeed it is one of the points we wish to emphasise. If it be replied that such a condition is an essential feature of these shows, then all we can say is that our warnings are fully justified. What Mr. Fowell says about the churches and the crockery may be quite true, but he does not whiten his own particular kettle by calling somebody else's pot black.—Ed. The Hospital.]

